# Neonatal Aeromedical Evacuation During COVID-19: An Interview With Captain Danielle James

**DOI:** 10.1093/milmed/usab250

**Published:** 2021-09-01

**Authors:** Danielle James, Laura A Talbot

**Affiliations:** 18th Healthcare Operations Squadron, Kadena AB, Okinawa 96368, Japan; Department of Neurology, College of Medicine, University of Tennessee Health Science Center, Memphis, TN 38163, USA

## Abstract

During the coronavirus-19 pandemic, limited information existed about the risks and consequences of severe acute respiratory syndrome coronavirus 2 infection associated with maternal transmission to neonates. With rapidly evolving evidence, Air Force Neonatal Intensive Care Unit nurses at U.S. Naval Hospital Okinawa, Japan, adapted their standard operating procedures to safeguard their at-risk neonatal patients. This interview describes an Air Force NICU nurse’s view of neonatal transport and nursing care during the coronavirus-19 pandemic.

## INTRODUCTION

In late 2019, a new coronavirus emerged. Severe acute respiratory syndrome coronavirus 2 (SARS-CoV-2) spread worldwide, with over 112,000,000 individuals testing positive for coronavirus-19 (COVID-19) and contributing to over 2.4 million deaths by March 14, 2021.^1^ Healthcare teams had to adapt quickly at a time when evidence and recommendations were continually evolving, including care for pregnant women, their fetuses, and the neonates. Initially, reports were based on an accrual of anecdotal cases and a select population of hospitalized patients or those seeking acute care. The risk of a vertical transmission from a COVID-19-positive mother to her infant was unknown. However, emerging cases in late May 2020 showed that 3.1% of 311 babies born to mothers with COVID-19 were positive within a week of birth, with no deaths and only one intensive care unit case.^2^ Limited information about the consequences of SARS-CoV-2 infection existed for this population.^3^ Nevertheless, it quickly became clear that pregnant women and neonates would require special considerations regarding the prevention, diagnosis, and management of COVID-19. With evidence evolving rapidly, an abundance of caution was advised as knowledge accumulated. Although unknown aspects remain, infants who test positive for SARS-CoV-2 tend to exhibit mild to no symptoms of COVID-19 and severe illness is rare.^4^ At a greater risk for severe illness are infants who have been born prematurely and those infants with underlying medical conditions.

With the current limited availability of vaccine against SARS-CoV-2, low vaccination rates where the vaccine is available, and vaccination of children under the age of 16 years not yet approved, many countries and territories worldwide have implemented strategies to maintain social distancing and minimize interaction to reduce transmission of this coronavirus.

U.S. Naval Hospital Okinawa (USNHO), Camp Foster, Japan, is home to the only DoD Level III Neonatal Intensive Care Unit (NICU) for the U.S. Indo-Pacific Command (USINDOPACOM). This Level III NICU maintains a state of training and readiness to provide premier neonatal care to neonates of service members and DoD beneficiaries stationed in the western Pacific (WESTPAC). Infants in Level III NICU require continuous monitoring of respiration and/or heart rates by apnea monitor, pulse oximeter, or transcutaneous monitors, with the availability of mechanical ventilation and incubators for life-saving support. This NICU specialty team provides theater-wide neonatal consultation, educational outreach, care, and aeromedical evacuation services. This interview describes a member of this team Capt Danielle James’ perspective of neonatal transport and nursing care provided by Air Force (AF) NICU nurses during the COVID-19 pandemic.

Aeromedical transportation has been utilized for decades to move patients across the world for medical care and treatment, with approximately 2% of patients transported by specialty teams such as neonatal teams.^5^ Unique to aeromedical missions, Kadena Air Base in Okinawa, Japan, is home to the only neonatal critical care air transport team that carries neonates to a higher echelon of care for this geographical region. This team is comprised of four physicians, four nurses, and two medical technicians who provide care for those serving the U.S. INDOPACOM region, supporting more than half a million DoD service members and their families. Throughout the mission, the neonatal transport team collaborates with the aeroevacuation team and flight crew. During the global COVID-19 pandemic, this specialized team has provided critical care transport to 36 infants and their families, traveling over 86,000 miles from both U.S. overseas medical treatment facilities and host nation medical facilities to NICUs across the CONUS. During the height of the COVID-19 pandemic, neonates were aeromedically evacuated from South Korea, Guam, and Japan. The neonatal transport team adhered to strict guidelines for personal protective equipment (PPE) and restriction of movement (ROM) mitigation plan to prevent the transmission of SARS-CoV-2. Through the implementation and practice of these rigorous measures, the NICU transport team, patients, and families have experienced no COVID-19 cases or conversions of COVID-19 test to positive despite traveling in disease “hot-spots” during the height of the pandemic. While we believe that the safe practices implemented above were important, we have no evidence that they prevented COVID-19 conversions.

## Interview Questions for NICU Transport Nurse, Captain Danielle James, during the COVID-19 Pandemic

***Could you provide information on your military career and your position/role during the COVID-19 pandemic?***

From 2019 to the present, I have served as an active duty NICU nurse in the U.S. AF, stationed at Kadena Air Base, Okinawa, Japan. My role is team leader in the Level III NICU where I provide nursing care and annually attend over 80 high-risk deliveries. As a member of the NICU transport team, I am one of the four nurses who provide NICU transport to all of the USINDOPACOM region. I have led 12 missions and managed over 170 inflight patient care hours. I have been a nurse since 2014, practicing in the critical careenvironment.

The AF has an NICU transportation course on Kadena Air Base, Japan, that focuses on the roles and responsibilities necessary when transporting an infant patient and what is essential to carry out the mission successfully. ***Can you comment on it? How did it prepare you for your current position?***

The NICU transport course focuses on teaching healthcare professionals’ aerophysiology and equipment. NICU aeromedical transport personnel consists of a neonatologist, NICU nurse, and NICU technician. In accordance with local policy and procedure, all NICU transport team members must complete the transport training course and several training missions. The NICU Transport Team Coordinators are responsible for providing all NICU personnel with job-specific inflight training. All NICU transport team members undergo monthly simulations with equipment, patient care scenarios, and biannual static transport simulations to maintain currency requirements.

***How would you describe what it was like caring for patients as an AF nurse during the COVID-19 pandemic? What situations proved most challenging in providing care for patients during the pandemic?***

Throughout the COVID-19 pandemic, the NICU in Okinawa has had to implement new policies and procedures to safeguard our fragile patients, families, and staff. One crucial policy was strict visitation practices that significantly restricted the number of personnel and visitors into the NICU. Parents could not enter the NICU while waiting for their own COVID-19 test results, so U.S. Naval Hospital Okinawa set up a webcam on a secure network, allowing mothers to view their infant 24/7. Further, NICU personnel were frequently tested for COVID-19, isolated on ROM, and required to utilize PPE to avoid transmission of COVID-19. Before transport, each patient and their accompanying parents were tested to confirm COVID-19-negative status. As we transport to and from high-risk COVID-19 regions, our neonatal transport team donned PPE, including N95 respirators, face shields, gloves, and gowns when interacting with local medical teams at both pickup and accepting facilities. Once the team arrived at the drop-off destination, we followed a strict ROM mitigation plan to reduce exposure risk.

**FIGURE 1.**
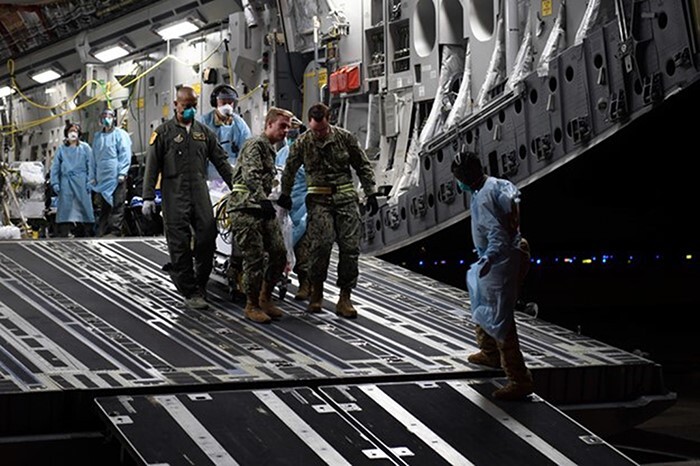
During a Neonatal Intensive Care Unit (NICU) aeromedical evacuation mission, Air Force (AF) medical personnel move an infant patient off a C-17 Globemaster III (U.S. AF photo by Staff Sgt Jason Huddlestor, VIRIN: 200330-F-GO352-1036, courtesy of the media defense.gov).

Source: https://www.dvidshub.net/image/6156180/ccatt-transport-neonatal-patients, accessed June 20, 2021.

The 18th Aeromedical Evacuation Squadron (AES) provides aeromedical evacuations for 540,000 beneficiaries and covers the largest area of responsibility (AOR) for the Pacific theater, spanning 100 million square miles.^6^ The 18th AES along with their partners in the 909th Air Refueling Squadron (ARS) maintain a 24-hour alert for emergencies outside the weekly patient pickup schedule, including the NICU transport (Fig. 1). The KC-135 Stratotanker from the 909th ARS is the airframe most often seen in the media, although any available aircraft can be configured to carry patients, including the C-17, C-130, or KC-10.^6,7^***How many patients are typically present in the aircraft during transport of the neonate? Has this changed since the COVID-19 pandemic started? Is it only the newborn and mother or are there others besides the medical and aircrew personnel on the flight?***

**FIGURE 2.**
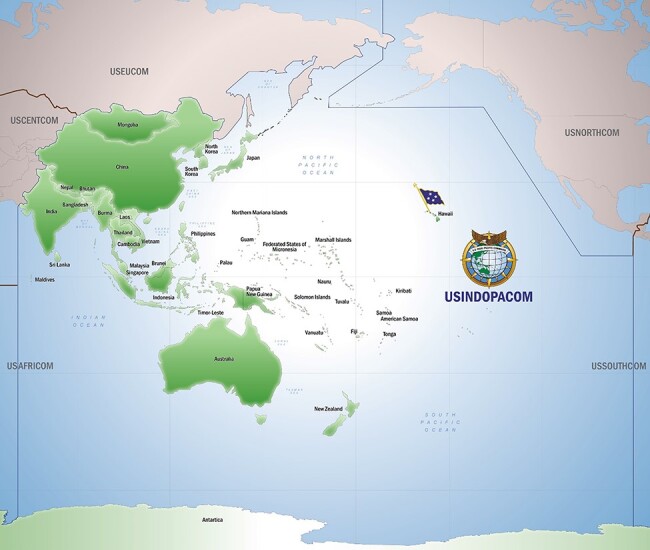
The U.S. INDO-Pacific Command (USINDOPACOM) area of responsibility. Courtesy of defense visual information distribution service. Source

For the most part, there are restrictions on all space A travel and the only additional passengers on flights have been patients (mothers and neonates). Every flying squadron has their own guidance for transport. Even on a flight where all teams are from Kadena, each flying squadron may have different requirements and specific restrictions to follow when at the destination location. As pointed out in the question, the geographical area of the INDOPACOM region (Fig. 2) encompasses over half the world’s surface. The diversity of the Asia-Pacific region spans to 36 nations, with over 3,000 different languages and comprises over half of the world’s populace.

https://www.pacom.mil/About-USINDOPACOM/USPACOM-Area-of-Responsibility/, accessed June 20, 2021.

Limiting cross-contamination of the crew is critical and can be a challenge in close quarters of an aircraft, especially during COVID-19.^8^ AE, depending on the aircraft and its staff complement, typically has specific procedures to limit cross-contamination. Can you give an example of the types of airplanes used during neonatal transport and how the separation of a “hot” zone (contaminated area with patients), a transition zone, and a “cold” zone (uncontaminated area for crew) has been used during the pandemic? If possible, please specify any modifications that are associated with flight duration and boarding procedure during COVID-19.

**FIGURE 3.**
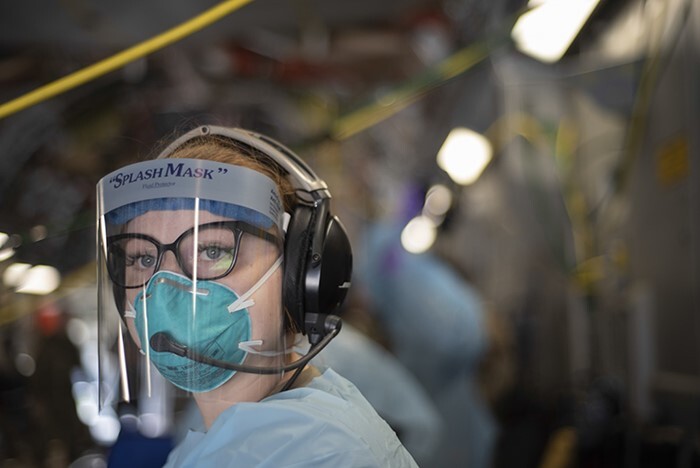
A member of the 18th Aeromedical Evacuation Squadron from Japan’s Kadena Air Base, one of the few neonatal critical air transport teams in the world. Personal protective equipment worn during a Neonatal Intensive Care Unit transport (U.S. Air Force photo by Staff Sgt James Miller, VIRIN: 200330-F-IP058-435, courtesy of the media defense.gov).

Source: https://www.dvidshub.net/image/6155094/twin-newborns-evacuated-amidst-covid-19-crisis, accessed April 8, 2021.

I have not personally flown on a mission with a COVID-19-positive patient. I do know that AE has developed a contamination “box” in which a patient who is COVID-19-positive can fly without risking exposure to the rest of the aircrew, but I do not have specifics on this equipment. Figure 3 shows a member of the 18th AES during an NICU transport mission.

***What type of care is given to the mother and neonate during transport? Can you talk about the modular NICU unit used in flight and is it the same unit that is used in the NICU?***

**FIGURE 4.**
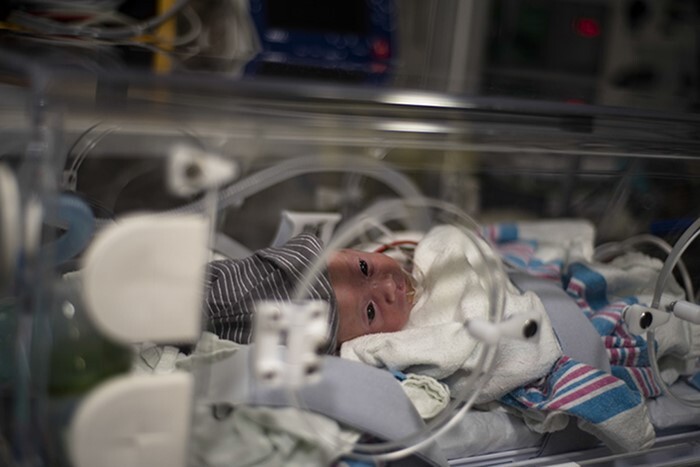
Actual Neonatal Intensive Care Unit patient transport from Osan Air Base, Korea, to Walter Reed National Military Medical Center, Maryland, USA (U.S. Air Force photo by Staff Sgt James Miller, VIRIN: 200330-F-IP058-1002, courtesy of the media defense.gov).

Source: https://www.dvidshub.net/image/6155027/twin-newborns-evacuated-amidst-covid-19-crisis, accessed April 8, 2021.

It is the goal of the Neonatal Transport Team to continue to provide family-centered care throughout air transport. In preparation for the flight, neonatologists establish early and frequent communication with the NICU case manager and parents to ensure that all the necessary personal documents are procured before the flight. During this time, the parents are briefed on the transport date and time, baggage, aircraft, in-flight family-centered care, and information on the receiving NICU. One key component to providing family-centered care is allowing the mother to continue to utilize a breast pump or to breastfeed the infant if it is safe for her to do so. Before the flight, parents are asked about the breast pump to be utilized, to ensure that it has been tested and is safe to use inflight; if not, the NICU at USNHO provides mothers with a hand breast pump. Breast milk storage information is also provided to the parents, and the Neonatal Transport Team is equipped to appropriately store and utilize breast milk inflight. Establishing frequent and good communication with the parents allows a better understanding of their capabilities and roles throughout all air transport stages. Every mission’s goal is to continue to have the parents directly involved in the patient’s care. In all aircraft, the parents can participate in hands-on care if the infant is stable and it has been agreed upon by all NICU transport team members. Each neonatal transport is unique and, based on the patient’s stability and needs, may limit the parent’s involvement in care, similar to the situation in the NICU. One of the most significant limitations with parents’ involvement is the temperature variability inside the aircraft, which often limits the amount of time that the infant can have portholes on the Neonatal Transport System open or be out to have feedings with parents and be held. Figure 4 shows an NICU infant patient in the neonate transport system during an NICU aeromedical evacuation mission.

***Can you describe the conditions in the Aeromedical Evacuation system when caring for patients with COVID-19? What types of care did these patients need? What precautions were taken during patient transport missions?***

**FIGURE 5.**
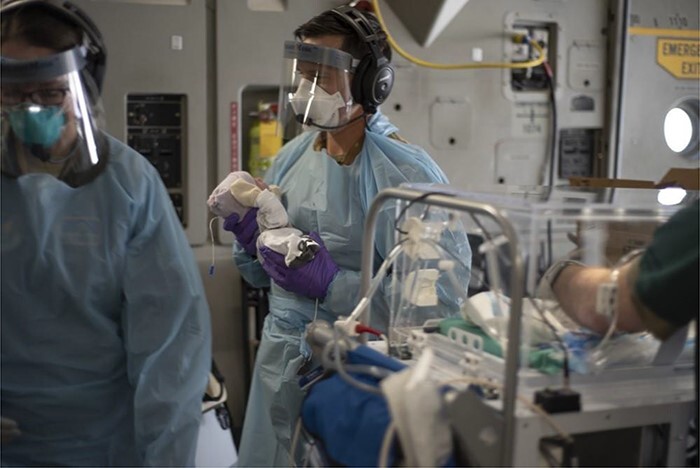
Personal protective equipment worn during a Neonatal Intensive Care Unit transport (U.S. Air Force photo by Staff Sgt James Miller, VIRIN: 200330-F-IP058-798, courtesy of the media defense.gov).

Source: https://www.dvidshub.net/image/6155090/twin-newborns-evacuated-amidst-covid-19-crisis, accessed April 8, 2021.

Since the start of the pandemic, the neonatal transport team has not had a COVID-19-positive patient or family member, despite traveling to high-risk countries like South Korea, Japan, and Guam and delivering these patients across the USA. Preserving compliance with USNHO NICU policy, parents must be tested for COVID-19 and show negative results before transport. This policy further requires parents to utilize facemasks throughout all phases of transport. Inflight Neonatal Transport Team members must wear an N-95 mask at all times, along with standard contact PPE. When the Neonatal Transport Team interacts with Emergency Medical Services for ground transport and the receiving facility, the team must use droplet PPE precautions (eye protection, N-95 mask, gown, and gloves) (Fig. 5). Once care has been assumed by the receiving facility, parents must have another COVID-19 test with negative results and ROM for 7-14 days in a hotel before being allowed to enter the NICU. Adherence to the PPE protocol and ROM mitigation plan has prevented the NICU transport team, patients, and families from contracting the virus.

***Has the COVID19 pandemic influenced your clinical practices and how you care for patients?***

The neonatal population is susceptible to infections, specifically respiratory illnesses; however, the COVID-19 virus does not affect neonates the same way as adults. The NICU in Okinawa, Japan, has not had a COVID-19-positive patient. However, during the pandemic, the NICU’s focus has been to keep our patient population safe while still providing parents access to participate in their infant’s care to promote bonding. Parents are educated on the facilities’ standards and policies regarding social distancing, masking, hygiene, and travel restrictions to minimize the risk of COIVD-19. The utilization of rapid testing has allowed parents to travel with their infant during transport and NICU entry to outside facilities. Finally, the integration of video conferencing on a secure network has been made available when in-person visitation is not possible. Being located on a remote island in the Pacific has dramatically impacted the import of supplies. It takes on average 2-4 months longer to obtain supplies than it did pre-pandemic. To reduce waste, the unit has implemented an on-shift PPE tracker and supply kits such as intravenous start and laboratory kits to minimize the supplies wasted. Tracking supplies and recognizing when inventory is low has been essential to maintaining our mission.

### Lessons Learned: Experiencing the Unexpected

***What were the most valuable lessons you learned during COVID-19 that may benefit future nurses faced with similar issues in a pandemic?***

The most valuable lesson learned from being an NICU nurse during COVID-19 is to be adaptable. As pandemic infection rates fluctuate globally, military members and their families must adhere to the Command Staff Directive, along with local hospital policies and guidance. COVID-19 has affected patient care, access to supplies, and staffing. Being adaptable allowed the NICU to continue to excel at both missions and to deliver high-quality patient-centered care.

As military nurses, we are consistently trained for the “what if” and to respond to the unexpected. Everything we do is centered on readiness. It is part of the AF Comprehensive Medical Readiness Program (CMRP). The CMRP is a clinical skills sustainment program used to ensure the currency of medical personnel through Sustained Medical Readiness Training. The CMRP divides medical readiness training into categories to support targeted application for specific specialties, personnel, and missions across the U.S. AF medical operations.

The medical aspects of the air expeditionary force center around the three missions that military personnel prepare: wartime, humanitarian, and disaster response, which includes pandemics. The military has practiced a pandemic disaster response plan and performed exercises in preparation for the possibility of a pandemic such as COVID-19.

Regarding the USAF Nursing Continuum of Care, AE transport focuses on the evacuation of patients to the next level of care using helicopters or fixed-wing or motor vehicles. Specialty units such as neonatal transport require specialized training, particularly because being taken up to altitude affects the patient’s condition and care. Crucial is the joint readiness with AE and the NICU during these transport missions. Neonatal AE transport occurs at various strategic locations, including Brook Army Medical Center, USNHO, and Landstuhl Regional Medical Center, with each having CONUS/OCONUS (outside the CONUS) capabilities for NICU transport.

## CONCLUSION

Air Force NICU nursing at USNHO is unique in that we provide neonatal care to infants of service members and DoD beneficiaries stationed in the WESTPAC, including NICU transport for all of INDOPACOM. It is a demanding subspecialty of nursing that requires a high degree of skill, dedication, and emotional strength. There are high expectations and personal commitments that compete with family and off-duty time. On reflection, most AF NICU nurses maintain that it is the most rewarding experience in their professional career. The opportunity to be a part of history during a worldwide pandemic, to provide care and transport to American service members and their families during extreme need, and to accomplish the mission safely despite the circumstances and personal cost is an unparalleled experience and one that holds tremendous pride.
